# Coronary heart disease and chronic obstructive pulmonary disease prevalence and temporal trends among United States adults: a national population-based study

**DOI:** 10.3389/fepid.2026.1840095

**Published:** 2026-06-25

**Authors:** Muinat Abolore Idris, Valerie M. Valenzuela, Juan Aguilera

**Affiliations:** 1 Department of Health Promotion and Behavioral Sciences, School of Public Health, The University of Texas Health Science Center at Houston, Houston, TX, United States; 2 Department of Environmental & Occupational Health Sciences, School of Public Health, The University of Texas Health Science Center at Houston, Houston, TX, United States

**Keywords:** chronic obstructive pulmonary disease (COPD), co-morbidity, coronary heart disease (CHD), disparities, linear trend

## Abstract

**Background:**

Chronic obstructive pulmonary disease (COPD) and cardiovascular disease (CVD) are two independent leading causes of morbidity and death, with co-existence contributing substantially to morbidity and deaths among aging populations. Despite growing evidence on the impact of COPD-CVD co-existence, little is known about COPD-CHD co-morbidity, as other previous studies mostly examine CVD as a single aggregated health outcome, obscuring disease-specific trajectories.

**Objective:**

To assess 5-year national trends and sociodemographic disparities in the prevalence of coexisting CHD-COPD co-morbidity.

**Methods:**

Using the 2020−2024 National Health Interview Survey Integrated Public Use Microdata Series adults' dataset, we estimated the national prevalence of CHD-COPD co-morbidity. Weighted descriptive statistics, multivariate regression models, and sensitivity analysis were used to examine the associations.

**Results:**

CHD-COPD co-morbidity prevalence showed no statistically significant increase from 2020 to 2024; although the estimated linear trend was positive, it did not reach statistical significance (OR per 1-year increase = 1.04; 95% CI: 0.997–1.08; *p* = 0.072). Age, sex, body mass index, race/ethnicity, geographical region, smoking status, insurance status, income, and educational attainment were strong independent predictors of CHD-COPD co-morbidity. Older age (aOR: 1.004; 95% CI: 1.003–1.004, *p* < 0.0001) was independently associated with higher odds; current and former smokers had approximately 5–6 times higher odds of CHD-COPD co-morbidity.

**Conclusion:**

CHD-COPD co-morbidity is a rare but clinically significant condition, with a stable adjusted prevalence of approximately 0.35%–0.43% annually among United States adults from 2020 to 2024. Smoking, older age, male sex, lower income, and Southern residence were the strongest independent predictors of CHD-COPD co-morbidity. These findings underscore the need for integrated, sociodemographically targeted prevention and chronic-disease management strategies addressing shared cardiopulmonary risk factors, particularly tobacco use, across vulnerable populations.

## Background

Coronary heart disease (CHD) and chronic obstructive pulmonary disease (COPD) are major causes of morbidity and mortality nationally and globally, and rank among the top ten leading causes of death (1). COPD is a progressive respiratory disease characterized by restricted airflow and respiratory symptoms (2). Beyond pulmonary impairment, COPD is increasingly recognized as a systemic disease associated with multiple co-morbidities, substantial symptom burden, and premature mortality among the aging population (3–6). Among these co-morbidities, cardiovascular diseases (CVDs) are particularly common. Adults with COPD are more than twice as likely to have at least one cardiovascular condition and face an elevated risk of multiple chronic diseases (7, 8).

Beyond cross-sectional associations, growing and accumulating evidence shows that COPD is frequently associated with the development and onset of other chronic diseases. Large registry and co-morbidity-network studies reported that COPD clusters with several chronic diseases, contributing to complex multimorbidity patterns and increased healthcare burden (8–11). These patterns likely reflect shared underlying mechanisms, as COPD and CVD involve overlapping biological pathways and interact across acute and chronic disease processes, contributing to increased cardiovascular burden among individuals with COPD. In the United States, approximately 16 million adults have been diagnosed with COPD, underscoring the substantial population burden of COPD-related co-morbidities (1). Furthermore, a national mortality study reported that although age-adjusted mortality involving both COPD and CVD declined through 2009 and remained relatively stable through 2018, rates increased pronouncedly during 2018–2020, highlighting the ongoing public health significance of cardiopulmonary multimorbidity and persistent sociodemographic disparities (12, 13).

Despite the extensive research linking COPD and cardiovascular disease, most studies examine CVD as a single aggregate health outcome, potentially obscuring disease-specific trajectory and mechanism associations. Studying coronary heart disease (CHD) away from the broader CVD umbrella is critical in epidemiological and clinical research, as CVD comprises heterogeneous conditions with different mechanisms, and CHD differs from other cardiovascular conditions in etiology, pathophysiology, prevention, and clinical management. CHD is primarily driven by atherosclerosis and systemic inflammation; other cardiovascular conditions in COPD are often linked to hypoxemia and pulmonary vascular changes (14, 15). Therefore, combining heterogeneous cardiovascular outcomes into a single aggregate category may obscure important epidemiologic patterns, limit precise risk stratification, and opportunities for targeted prevention management strategies.

Although systematic reviews and meta-analyses consistently report strong associations between COPD and CVD, CHD is rarely isolated and examined as a distinct pathophysiological cardiovascular outcome (16, 17) or examined temporal trends in CHD-COPD co-morbidity at the population level. Addressing this gap is important given that CHD accounts for approximately one in three deaths in the United States (18), and individuals with both CHD and COPD conditions experience greater healthcare utilization, poorer health outcomes, and higher economic burden than those with either condition alone (14, 15, 19).

To address this gap, this study assessed 5-year national trends and sociodemographic disparities in the prevalence of CHD-COPD co-morbidity among United States adults using the National Health Interview Survey Integrated Public Use Microdata Series (NHIS-IPUMS) Sample Adult data (20). Understanding the temporal patterns and population disparities in CHD-COPD co-morbidity may help inform surveillance efforts, identify high-risk populations, and support targeted chronic disease prevention and management strategies (21).

## Methods

### Data source

This repeated cross-sectional study used pooled data from the 2020–2024 NHIS-IPUMS Sample Adult files, as indicated by the NHIS sample-adult flag, and restricted the analysis to adults with a valid record representing the national civilian population. The 2020−2024 survey contains 188,419 adult samples; of these, we excluded 37,567 observations with missing Sample Adult identifiers, leaving 150,852 adult samples for analysis. The IPUMS datasets were created by the Minnesota Population Center (20). The dataset is obtained from the NHIS, an annual, nationally representative, cross-sectional survey that monitors the health of the United States noninstitutionalized civilian population.

### Measured variables

**Coronary heart disease (CHD)** and **chronic obstructive pulmonary disease (COPD)** were defined using self-reported physician diagnoses from NHIS-IPUMS (20) and were categorized as binary variables (Yes/No). Participants who responded “Yes” to the question, “*ever told had CHD,”* were classified as having CHD, and those who responded “Yes” to the question, “*ever been told you had COPD,”* were classified as having COPD.

**CHD–COPD co-morbidity**, a composite variable was created to identify respondents with both COPD and CHD diseases i.e., participants reporting both conditions. Respondents with diagnosed COPD and those with diagnosed CHD at the time of the survey were classified as having COPD-CHD co-morbidity. This co-morbidity reflects the co-occurrence of both conditions at the time of assessment rather than the participant's lifetime history.

In addition to these dependent outcome variables, independent variables such as sociodemographic variables including age, sex, race/ethnicity, educational attainment, poverty level, insurance status, smoking status, body mass index (BMI), and region were included in the analysis. Race/ethnicity and educational attainment were recoded into five categories each. Respondents' smoking status was categorized into three groups: never smokers, former smokers, and current smokers. Respondents who reported “*being a current smoker, current every-day smoker, current some-day smoker, or provided an unclear smoking frequency*” were classified as current smokers. Poverty level was defined as the ratio of family income to the federal poverty threshold, and it was recoded into a binary variable (<1.0 = below threshold; ≥1.0 = at or above threshold), representing the poverty status, as used in the NHIS survey. These covariates were selected based on NHIS documentation of core content and prior studies on trends and disparities. Potential mediators, including physical activity, alcohol use, hypertension, and diabetes, were not included to avoid overadjustment bias.

### Statistical analysis

Survey-weighted descriptive statistics were used to characterize the study population. Crude annual prevalence of CHD-COPD co-morbidity was estimated using survey procedures. Adjusted annual prevalence and 95% confidence intervals (CIs) were estimated from survey-weighted logistic regression models treating survey year as a categorical variable and calculating predictive margins averaged over the covariate distribution. Secondly, separate survey-weighted multivariable logistic regression models were fitted for CHD, COPD, and CHD-COPD co-morbidity, respectively, to identify covariates associated with each dependent health outcome (CHD, COPD, and CHD-COPD co-morbidity). A third model was used to evaluate the presence of a monotonic temporal trend; temporal trends were evaluated using survey-weighted logistic regression models with survey year modeled as a continuous variable, estimating the annual change in odds per one-year increase. A linear trend was assumed, given the relatively short study period, and to provide a parsimonious summary of changes over time. This assumption was formally assessed using a likelihood ratio test comparing models with year specified as a continuous versus categorical variable, which indicated no evidence of deviation from linearity (*p* > 0.05). To determine the robustness of the study, sensitivity analyses comparing alternative specifications of year and age were done for CHD-COPD. Consistency between the categorical and continuous age and year models was used to evaluate the robustness of temporal findings. The model fit and inferences were evaluated based on statistical significance, consistency of effect estimates, and likelihood ratio tests of model fit.

Multicollinearity among covariates was assessed using variance inflation factors (VIFs) based on the fully adjusted CHD-COPD model, and all variables showed VIF values below five, indicating no substantial multicollinearity evidence. All estimates for the NHIS IPUMS complex survey design were accounted for by incorporating the sample weights (SAMPWEIGHT), variance estimation (PSU), and stratification (STRATA). Following NHIS recommendations, pooled Sample Adult weights were divided by five to rescale the sample weights and generate average annual population estimates. Missing data for CHD (0.29%) and COPD (0.13%) were minimal; therefore, complete-case analysis was performed. All analyses were conducted using SAS Version 9.4 (SAS Institute, Cary, NC), and statistical significance was defined as *p* < 0.05.

## Result

**Figure 1:**
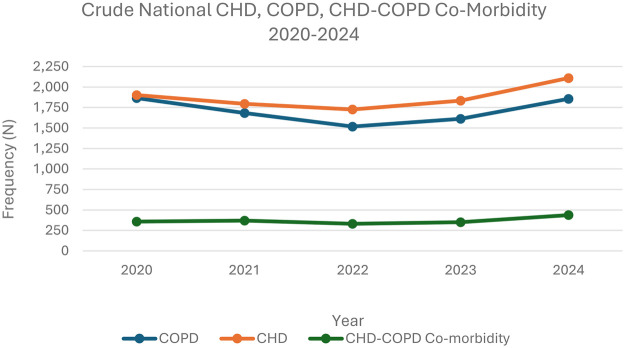
Crude survey-weighted national annual CHD, COPD, and CHD-COPD Co-morbidity among United States Adults (2020−2024).

Across 2020–2024, the crude, survey-weighted prevalence of COPD and CHD was low and stable at approximately 4.2−4.9% and 4.6−5.0%, respectively, per year, with a modest and statistically significant difference across years for COPD (*p* = 0.0006) (Table 1), which can also be visualized in Figure 1. CHD-COPD co-morbidity was rare, with approximately 0.83%–0.97% per year.

**Table 1:** Crude survey weighted annual prevalence estimates for COPD, CHD, CHD-COPD Co-morbidity among United States adults, IPUMS 2020–2024.

	COPD				CHD			
Year	Unweighted (*n*)	Weighted (*n*)	%	95% CI	Unweighted (*n*)	Weighted (*n*)	%	95% CI
2020	1,865	2,508,862	4.98	4.66–5.30	1,901	2,339,488	4.65	4.37–4.93
2021	1,683	2,340,012	4.63	4.35–4.91	1,796	2,489,402	4.93	4.65–5.21
2022	1,518	2,336,619	4.58	4.29–4.87	1,726	2,513,226	4.93	4.66–5.20
2023	1,612	2,222,171	4.31	4.03–4.58	1,833	2,482,580	4.82	4.57–5.07
2024	1,856	2,199.729	4.24	3.97–4.51	2,110	2,593,495	5.01	4.73–5.29
Total	8,534	11,607,394	4.54	4.38–4.71	9,366	12,418,192	4.87	4.72–5.02

	CHD-COPD co-morbidity			
Year	Unweighted (n)	Weighted (n)	%	95% CI
2020	357	420,329	0.83	0.72–0.94
2021	371	490,891	0.97	0.85–1.09
2022	331	463,092	0.91	0.79–1.02
2023	351	465,909	0.90	0.79–1.02
2024	437	496,559	0.96	0.85–1.07
Total	1,847	2,336,779	0.91	0.86–0.97

### Coronary heart disease (CHD) findings

**Figure 2:**
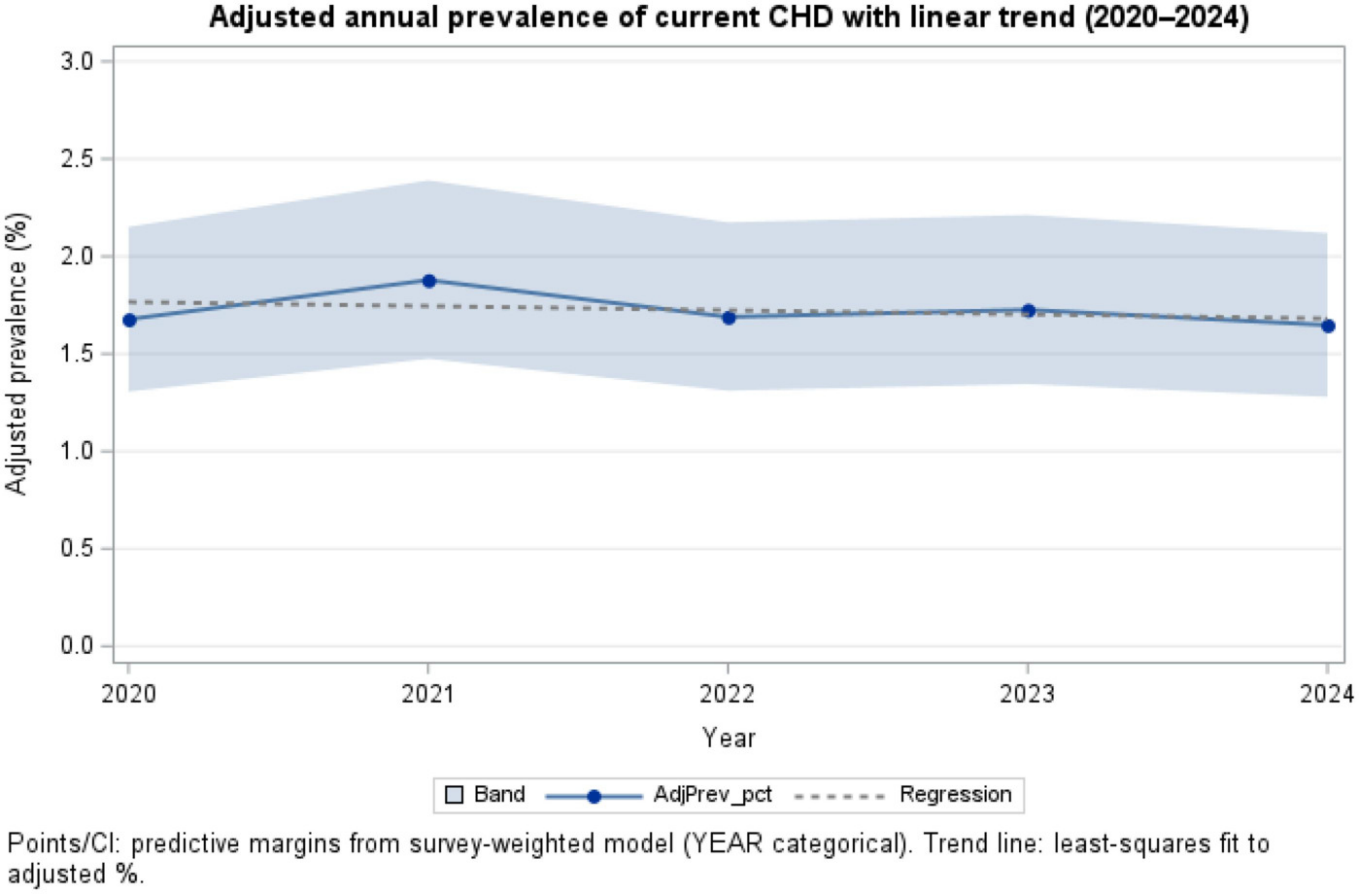
Adjusted annual prevalence of CHD (predictive margins) with model-implied linear trend. The dashed line shows the model-implied linear trend from a separate survey weighted- model with “YEAR” specified continuously (Year modeled as a continuous predictor).

Adjusted CHD prevalence was low and showed no statistically significant change across 2020–2024 (Supplementary Table S1). For the adjusted associations of CHD from a multivariable logistic regression model with year modeled as a categorical variable, year-specific adjusted odds ratios showed no significant temporal trend in CHD prevalence from 2020 to 2024 (Type 3 *p* = 0.50) (Table 2). Contrarily, covariates, including sex, smoking status, age group, insurance status, and lower income, showed a strong independent association with higher CHD odds (*p* < 0.0001), while higher educational attainment and residing in the West region were found to be associated with lower CHD odds (*p* < 0.05). Treating year as a continuous variable showed no evidence of a linear temporal trend in CHD (aOR per yea*r* = 0.98; 95% CI: 0.95–1.02; *p* = 0.49) (Table 3), a consistent finding with the categorical year model. Also, correspondingly, the adjusted CHD prevalence remained stable over time (1.65%–1.88%) (Figure 2), and the predictive margins closely align with the flat model-implied linear trend line.

**Table 2:** Survey-weighted logistic regression for adjusted associations with CHD, 2020−2024 (“YEAR” categorical model).

Variable	Comparison (vs. referent)	aOR	95% CI	*p* value
YEAR	2021 vs. 2020	1.122	0.959–1.312	0.1492
	2022 vs. 2020	1.007	0.860–1.180	0.9274
	2023 vs. 2020	1.029	0.882–1.202	0.7130
	2024 vs. 2020	0.982	0.836–1.154	0.8255
Sex	Male vs. Female	1.984	1.778–2.214	<0.0001***
Age category	Early vs. young adulthood	1.405	0.899–2.197	0.1356
	Late vs. young adulthood	11.426	7.379–17.691	<0.0001***
	Older vs. young adulthood	23.839	15.032–37.807	<0.0001***
BMI	Normal vs. Underweight	0.710	0.437–1.152	0.1653
	Overweight vs. Underweight	0.793	0.491–1.280	0.3424
	Obese vs. Underweight	1.058	0.660–1.696	0.8144
Race/ethnicity	AIAN vs. White	1.056	0.803–1.389	0.6940
	Asian vs. White	1.299	0.768–2.198	0.3283
	Black vs. White	1.021	0.869–1.200	0.7978
	Other vs. White	1.191	0.860–1.648	0.2923
Region	Midwest vs. Northeast	0.872	0.732–1.040	0.1276
	South vs. Northeast	1.112	0.948–1.304	0.1935
	West vs. Northeast	0.651	0.534–0.795	<0.0001***
Smoking status	Current vs. Never	1.751	1.521–2.017	<0.0001***
	Former vs. Never	1.669	1.483–1.878	<0.0001***
Education	High School/GED vs. < High School	0.967	0.818–1.143	0.6931
	Some College/AA vs. < High School	0.800	0.673–0.952	0.0119*
	Bachelor's degree vs. < High School	0.557	0.453–0.685	<0.0001***
	Graduate degree vs. < High School	0.549	0.442–0.681	<0.0001***
Insurance	Yes vs. No	2.183	1.687–2.824	<0.0001***
Income-to-poverty	Below threshold (<1.0) vs. ≥1.0	2.083	1.815–2.391	<0.0001***

Statistical significance: *p < 0.05, ***p < 0.0001. Race/ethnicity: “AIAN” = American Indian and Alaska Native, “White” = Non-Hispanic White, “Black” = Non-Hispanic Black, “Others” = “Mixed-race”. Age group: “young adulthood” = 18−24 years, “Early adulthood” = 25–44 years, “Late adulthood” = 45–64 years, “Older adulthood” = 65 + years. BMI: “Underweight” = <18.5 kg/m^2^, “Normal weight” = 18.5–24.9 kg/m^2^, “Overweight” = 25.0–29.9 kg/m^2^, “Obese” = ≥30.0 kg/m^2.^

**Table 3:** Survey-weighted logistic regression for adult adjusted CHD linear trend over time (“YEAR” continuous model), 2020–2024.

Variable	Comparison (vs. referent)	Adjusted OR	95% CI	F-value	*p*-value
YEAR (continuous)	Per +1 year	0.988	0.954–1.023	0.48	0.4904

### Chronic obstructive pulmonary disease (COPD) findings

**Figure 3:**
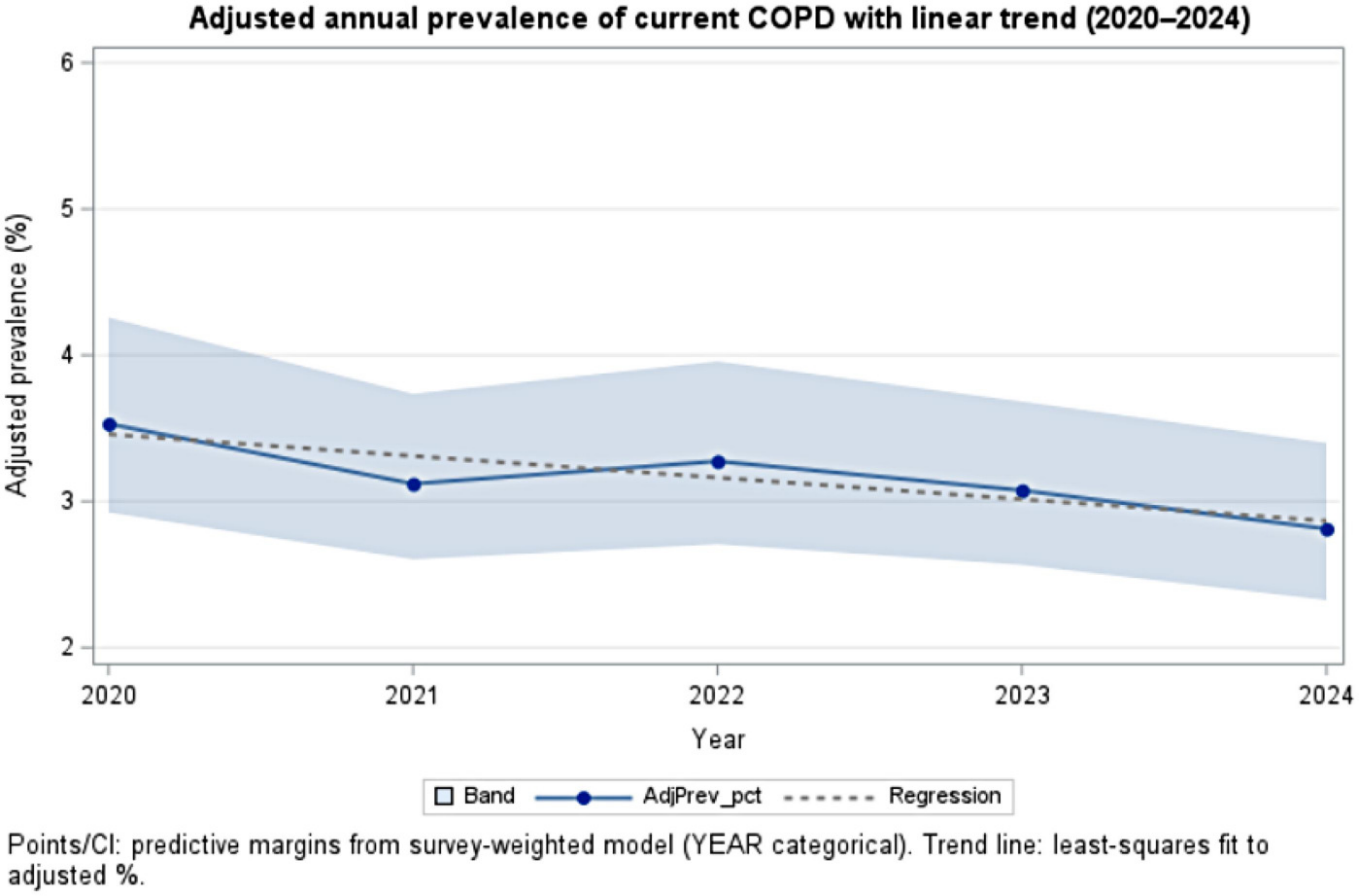
Adjusted annual prevalence of COPD (predictive margins) with model-implied linear trend. The dashed line shows the model-implied linear trend from a separate survey weighted- model with “YEAR” specified continuously (Year modeled as a continuous predictor).

The adjusted COPD prevalence declined significantly from 3.53% in 2020 to 2.81% in 2024, a reduction of approximately ∼20%, indicating a downward temporal trend (Supplementary Table S2; Figure 3). The adjusted associations of COPD from a multivariable logistic regression model with year modeled as a categorical variable show that year-specific COPD odds ratios relative to 2020 were small in magnitude, ranging from 0.79 to 0.93 (Table 4). Although statistically significant reductions were observed in 2023 (aOR = 0.87; 95% CI: 0.75–0.99) and 2024 (aOR = 0.79; 95% CI: 0.68–0.91), the overall year effect was modest but significant (F = 2.70; Type 3 *p* = 0.029). COPD was strongly associated with key covariates, including sex, smoking, race, age, BMI, insurance, income, educational attainment, and geographical region. Current and former smokers had substantially six times and three times higher odds of COPD, respectively, while risk increased with age and lower income. Adults residing in the Midwest and South also had higher odds of COPD. In contrast, male sex, Black and AIAN race/ethnicity, and higher educational attainment were associated with lower COPD odds. Treating year as a continuous variable, the linear trend model indicated a significant decline in adjusted COPD odds over time (OR per yea*r* = 0.953; 95% CI: 0.923–0.980; *p* = 0.003) (Table 5), a consistent finding with the categorical year model and the downward trajectory in the adjusted prevalence as shown in Figure 3, where predictive margins are overlaid with the model-implied linear trend. This implies that for each additional calendar year, the adjusted odds of COPD decrease by approximately 4.7% on average.

**Table 4:** Survey-weighted logistic regression for adjusted associations with COPD, 2020–2024 (“YEAR” categorical model).

Variables	Comparison (vs. referent)	aOR	95% CI	*p* value
YEAR	2021 vs. 2020	0.880	0.773–1.002	0.0541
	2022 vs. 2020	0.925	0.811–1.055	0.2466
	2023 vs. 2020	0.867	0.754–0.997	0.0451*
	2024 vs. 2020	0.791	0.684–0.914	0.0015**
Sex	Male vs. Female	0.637	0.583–0.696	<0.0001***
Age category	Early vs. young adulthood	1.335	0.964–1.849	0.0822
	Late vs. young adulthood	4.579	3.345–6.268	<0.0001***
	Older vs. young adulthood	7.192	5.093–10.156	<0.0001***
BMI	Normal vs. Underweight	0.496	0.372–0.662	<0.0001***
	Overweight vs. Underweight	0.464	0.348–0.621	<0.0001***
	Obese vs. Underweight	0.759	0.569–1.014	0.0616
Race/ethnicity	AIAN vs. White	0.377	0.258–0.549	<0.0001***
	Asian vs. White	1.145	0.838–1.565	0.3937
	Black vs. White	0.825	0.709–0.960	0.0131*
	Other vs. White	1.165	0.893–1.520	0.2590
Region	Midwest vs. Northeast	1.206	1.018–1.430	0.0307*
	South vs. Northeast	1.208	1.030–1.416	0.0202*
	West vs. Northeast	0.995	0.829–1.194	0.9567
Smoking	Current vs. Never	6.005	5.384–6.697	<0.0001***
	Former vs. Never	3.110	2.778–3.481	<0.0001***
Education	Some College/AA vs. < HS	0.636	0.550–0.736	<0.0001***
	High School/GED vs. < HS	0.673	0.586–0.773	<0.0001***
	Bachelor degree vs. < HS	0.336	0.280–0.403	<0.0001***
	Graduate degree vs. < HS	0.267	0.213–0.336	<0.0001***
Insurance	Yes vs. No	1.822	1.521–2.183	<0.0001***
Income-to-poverty	Below threshold (<1.0) vs. ≥1.0	2.240	2.004–2.504	<0.0001***

Statistical significance: *p < 0.05, **p < 0.001, ***p < 0.0001. Race/ethnicity: “AIAN” = American Indian and Alaska Native, “White” = Non-Hispanic White, “Black” = Non-Hispanic Black, “Others” = “Mixed-race”. Age group: “young adulthood” = 18–24 years, “Early adulthood” = 25–44 years, “Late adulthood” = 45–64 years, “Older adulthood” = 65 + years. BMI: “Underweight” = <18.5 kg/m^2^, “Normal weight” = 18.5–24.9 kg/m^2^, “Overweight” = 25.0–29.9 kg/m^2^, “Obese” = ≥30.0 kg/m^2.^

**Table 5:** Survey-weighted logistic regression for adult adjusted COPD linear trend over time (“YEAR” continuous model), 2020–2024.

Variable	Comparison (vs. referent)	Adjusted OR	95% CI	F-value	*p*-value
YEAR (continuous)	Per +1 year	0.953	0.923–0.984	8.90	0.0030

### CHD-COPD co-morbidity findings

For the CHD-COPD co-morbidity analysis, rather than modelling age as a categorical variable, age was modelled as a continuous variable to avoid sparse data and quasi-complete separation, and to improve model stability while preserving full adjustment for age. The predictive margins for annual adjusted CHD-COPD co-morbidity prevalence remained low and stable over time (0.35%–0.43%) (Supplementary Table S3).

#### Adjusted associations of CHD-COPD co-morbidity from a multivariable logistic regression model with modeled year as a categorical variable

Year-specific adjusted CHD-COPD odds ratios relative to 2020 were generally null, and the overall year effect was not statistically significant (Type 3 F = 1.85; *p* = 0.118) (Table 6). Although modest elevations were observed in the years 2021 and 2024, there was no consistent monotonic pattern, and these did not alter the overall inference. Specifically, CHD-COPD co-morbidity was strongly associated with sociodemographic and behavioral factors. Older age and smoking showed the strongest associations, with current and former smokers having approximately 5–6 times higher odds. Male sex, lower income earners, South region residents, and insured individuals were also associated with higher CHD-COPD odds, while higher educational attainment, AIAN, Black, and other race/ethnicity groups, and normal or overweight BMI were associated with lower CHD-COPD co-morbidity odds (*p* < 0.05).

**Table 6:** Survey-weighted logistic regression for adjusted associations with CHD-COPD co-morbidity, 2020–2024 (“YEAR” categorical model).

Variable	Category (vs. Reference)	aOR	95% CI	*p*-value
Year	2021 vs. 2020	1.244	1.039–1.490	0.017*
	2022 vs. 2020	1.142	0.939–1.390	0.184
	2023 vs. 2020	1.175	0.976–1.414	0.088
	2024 vs. 2020	1.245	1.037–1.494	0.019*
Sex	Male vs. Female	1.304	1.161–1.465	<0.0001***
Age (continuous)	Per 1-year increase	1.004	1.003–1.004	<0.0001***
BMI	Normal vs. Underweight	0.492	0.320–0.756	0.001**
	Overweight vs. Underweight	0.552	0.358–0.852	0.007*
	Obese vs. Underweight	0.776	0.504–1.195	0.250
Race	AIAN vs. White	0.445	0.246–0.803	0.007*
	Asian vs. White	1.229	0.795–1.901	0.352
	Black vs. White	0.660	0.536–0.812	<.0001***
	Other vs. White	0.647	0.427–0.982	0.041*
Region	Midwest vs. Northeast	1.142	0.923–1.412	0.221
	South vs. Northeast	1.220	1.004–1.483	0.046*
	West vs. Northeast	0.876	0.705–1.089	0.234
Smoking status	Current vs. Never	5.501	4.643–6.517	<0.0001***
	Former vs. Never	5.527	4.776–6.395	<0.0001***
Education	< High School vs. < HS	1.00	Reference	<0.0001***
	High School/GED vs. < HS	0.630	0.528–0.752	<0.0001***
	Some College/AA vs. < HS	0.533	0.443–0.642	<0.0001***
	Bachelor's vs. < HS	0.237	0.188–0.299	<0.0001***
	Graduate vs. < HS	0.246	0.183–0.329	<0.0001***
Insurance	Yes vs. No	8.312	5.102–13.544	<0.0001***
Income-to-poverty	Below vs. ≥ Threshold	1.954	1.668–2.289	<0.0001***

Statistical significance: *p < 0.05, **p < 0.001, *** *p* < 0.001. Race/ethnicity: “AIAN” = American Indian and Alaska Native, “White” = Non-Hispanic White, “Black” = Non-Hispanic Black, “Others” = “Mixed-race”. Age group: “young adulthood” = 18–24 years, “Early adulthood” = 25–44 years, “Late adulthood” = 45–64 years, “Older adulthood” = 65 + years. BMI: “Underweight” = <18.5 kg/m^2^, “Normal weight” = 18.5–24.9 kg/m^2^, “Overweight” = 25.0–29.9 kg/m^2^, “Obese” = ≥30.0 kg/m^2.^

#### Linear time-trend model for CHD-COPD co-morbidity with “year” treated as a continuous predictor

**Figure 4:**
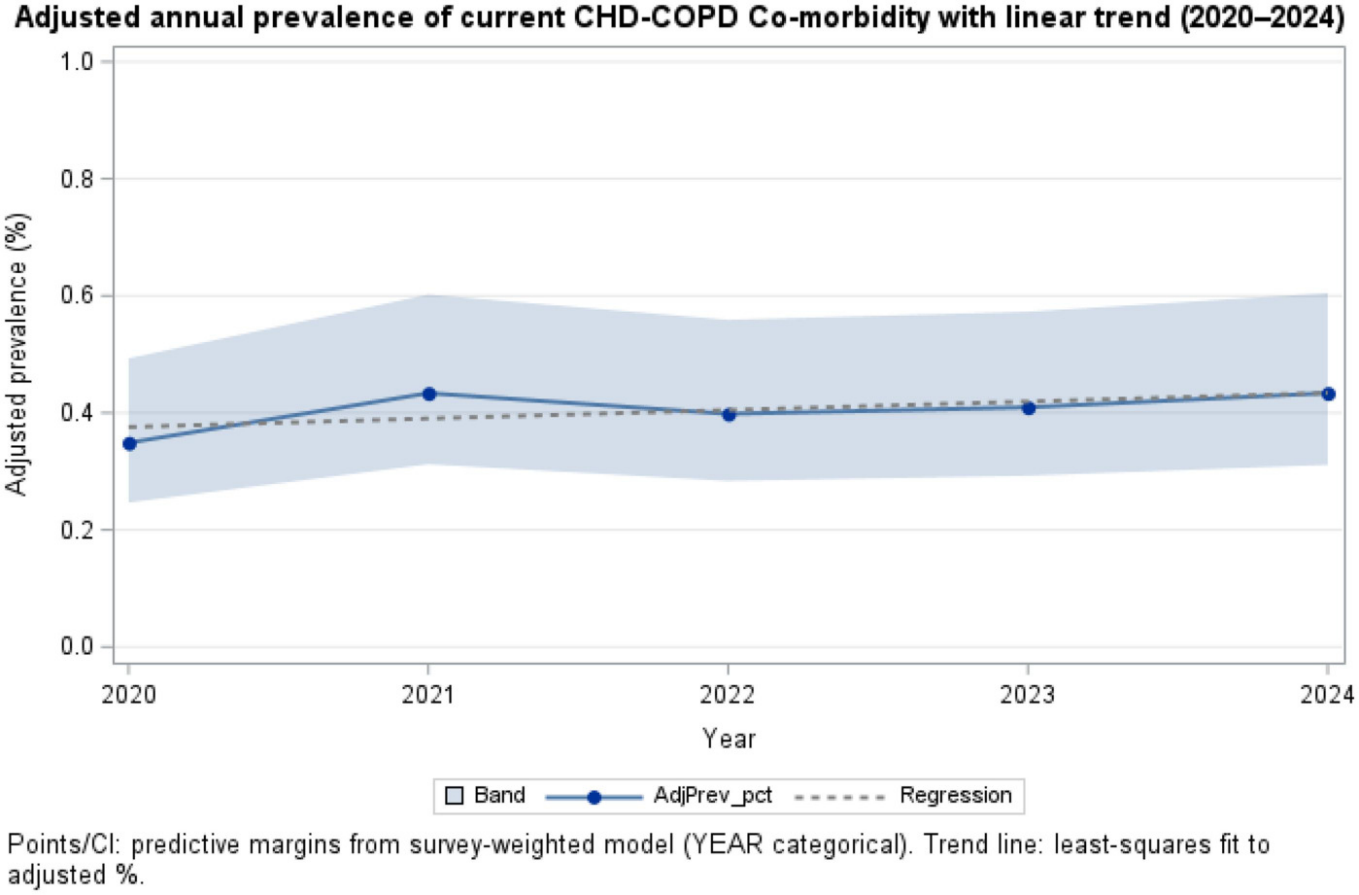
Adjusted annual prevalence of CHD-COPD co-morbidity (predictive margins) with model-implied linear trend. The dashed line shows the model-implied linear trend from a separate survey weighted- model with “YEAR” specified continuously (Year modeled as a continuous predictor).

There was no statistically significant linear trend in CHD–COPD co-morbidity from 2020 to 2024 (OR per yea*r* = 1.04; 95% CI: 0.997–1.08; *p* = 0.072) (Table 7). Although the point estimate suggested a modest increase, the confidence interval included the null, and the trend was not statistically significant. Consistently, the adjusted prevalence remained low and stable at approximately 0.30%–0.44%, with overlapping confidence intervals and an essentially flat model-implied trend in Figure 4. This implies that, while CHD-COPD co-morbidity is a rare health outcome, the adjusted odds of CHD-COPD co-morbidity increase by approximately 4% per additional calendar year.

**Table 7:** Adjusted association with CHD-COPD Co-morbidity outcome (survey-weighted logistic regression, YEAR continuous).

Variable	Category (vs. reference)	aOR	95% CI	F-value Type 3	*p*-value
YEAR (continuous)	Per + 1 year	1.038	0.997–1.080	3.25	0.072

### Sensitivity analysis

Findings for CHD-COPD co-morbidity were consistent across model specifications. Results were unchanged whether calendar year was modeled categorically or continuously, with no significant temporal trend observed in either specification (categorical: Type 3 F = 1.85, *p* = 0.118; continuous: OR per yea*r* = 1.04, 95% CI: 0.997–1.08, *p* = 0.072). Adjusted annual prevalence estimates were closely clustered with overlapping confidence intervals, and the model-implied trajectory in Figure 4 was essentially flat, indicating minimal year-to-year variation. Also, sensitivity analyses using alternative age specifications (categorical vs. continuous) yielded consistent results, with negligible changes in the magnitude, direction, or statistical significance of key covariate associations. Overall, there was no evidence of a statistically or clinically meaningful temporal change in CHD-COPD co-morbidity from 2020 to 2024.

## Discussion

This nationally representative analysis of NHIS-IPUMS data from 2020 to 2024 examined the prevalence of CHD-COPD co-morbidity and its individual components among United States adults. Our central findings are threefold: (1) CHD-COPD co-morbidity is a rare health condition that remained stable throughout the study period; (2) adjusted COPD prevalence declined modestly, whereas CHD prevalence remained unchanged; and (3) age, smoking status, sex, income, and geographic region were consistently associated with the three outcomes. By examining CHD as a distinct cardiovascular outcome rather than part of a broader CVD category, this study provides new population-level evidence on cardiopulmonary multimorbidity during and after the COVID-19 pandemic.

### CHD-COPD co-morbidity prevalence and temporal stability

The observed low prevalence and temporal stability of CHD-COPD co-morbidity may reflect a combination of the relatively short observation period, a genuine epidemiological plateau, and the underdiagnosis of one or both conditions. Prior studies have documented substantial undiagnosed cardiovascular disease, leading to under-recognition of the disease among individuals with COPD due to overlapping and shared symptoms, particularly dyspnea, which often clouds clinical attrition (22). Clinical and registry-based studies consistently report higher CHD prevalence among individuals with COPD than population-based surveys (6, 15, 23). Because NHIS relies on self-reported physician diagnoses, prevalence estimates likely represent conservative lower-bound estimates of the true burden, particularly among lower-income adults with limited healthcare access (24).

While our findings differ somewhat from national mortality analyses showing increased mortality involving COPD and cardiovascular disease during the COVID-19 period (12). Direct comparison is limited because the analyzed mortality rate and our findings for the self-reported disease prevalence capture different dimensions of disease burden. Therefore, our findings with the absence of a significant temporal increase in CHD-COPD co-morbidity prevalence may suggest stabilization following the acute pandemic-period surge disruptions, although longer observation periods are needed to confirm this pattern.

### Declining COPD prevalence and individual CHD stability

Adjusted COPD prevalence declined significantly between 2020 and 2024, whereas CHD prevalence remained stable. The COPD findings are broadly consistent with national surveillance reports showing either stable or declining prevalence in some population groups, particularly adults aged 18−44 years (25, 26). Also, the observed declined finding in our study may reflect an acceleration of previously observed downward trends in younger cohorts, cumulative effects of secular declines in smoking prevalence, particularly among younger adults, and improvements in disease prevention and management. Studies show that cigarette smoking cessation rates among United States adults have continued to increase, with evidence suggesting that declines in smoking prevalence are most pronounced in younger adults, which is expected to reduce the population incidence and ultimately the prevalence of COPD over time (27, 28).

In contrast, adjusted CHD prevalence showed a slight change in the evidence, consistent with prior studies indicating that recent declines in coronary disease burden have slowed. The combination of declining COPD prevalence and stable CHD prevalence may explain why CHD-COPD co-morbidity showed a non-significant trend. Given the rarity of the outcome and the limited five-year observation period, additional surveillance is needed to determine whether meaningful long-term trends exist.

### Smoking, age, and sex as key determinants

Smoking was the strongest modifiable predictor of CHD-COPD co-morbidity. Current and former smokers had substantially higher odds of co-morbidity than never smokers, a consistent finding with prior studies, as shared pathophysiology, including systemic inflammation, endothelial dysfunction, and oxidative stress, contributes simultaneously to both COPD progression and atherosclerotic cardiovascular disease (12, 17). Inflammatory mediators, including TNF-α, IL-6, and CRP, are elevated in smokers with COPD (14). Also, the observed elevated odds among former smokers likely reflect the long-term effects of cumulative tobacco exposure, including irreversible airflow obstruction and persistent cardiovascular residual risk from established atherosclerotic lesions (29). These findings underscore the importance of smoking prevention and cessation programs for reducing cardiopulmonary disease burden.

Increasing age was independently associated with higher odds of CHD-COPD co-morbidity, reflecting cumulative exposure to biological and environmental risk factors across the life course and the nature of cardiopulmonary damage accrual over the life course. Adults in the late adulthood (45–64 years) and older adulthood (65 + years) had substantially higher odds of both COPD and CHD (aOR = 4.58 and 7.19, respectively). This is similar to the NCHS Data Briefing, which reported that COPD prevalence increased steeply from 0.4% for adults aged 18–24 years to 10.5% for adults aged 75 years and older (25). Also consistent with previous studies, male sex was associated with higher odds of CHD-COPD co-morbidity and CHD, while females had higher COPD prevalence. This indicates the well-known sex-based differences in disease patterns, which likely reflect smoking history (higher lifetime smoking burden and more atherogenic lipid profiles historically observed in men) (15), and biological susceptibility and disease presentation (higher diagnosed COPD prevalence in women, particularly in younger and middle-aged cohorts) (3, 26).

### Socioeconomic and geographic disparities

Lower income and educational attainment were consistently associated with all three outcomes, highlighting persistent socioeconomic inequalities in cardiopulmonary health. Adults living below the federal poverty threshold had substantially higher odds of CHD-COPD co-morbidity, while higher educational attainment was protective. These findings align with national evidence demonstrating strong socioeconomic gradients in cardiovascular and respiratory disease conditions (30–37), underscoring the role of education as a social determinant of health. Education may influence disease risk through health literacy, occupational opportunities, and health behaviors (35), while the income-related disparities may reflect differences in smoking prevalence, environmental and occupational exposures, healthcare access, and treatment adherence (24, 34).

Regional differences were also evident. Residents of the South had higher odds of COPD and CHD-COPD co-morbidity, whereas adults in the West had lower CHD odds. These patterns mirror longstanding geographic disparities in chronic disease burden across the United States and may reflect differences in tobacco use, poverty, healthcare access, occupational exposures, and other social determinants of health (21, 31). The Midwest also showed elevated COPD odds consistent with industrial occupational exposure histories in that region.

### Race/ethnicity and insurance status

AIAN, Black, and other racial/ethnic groups had lower odds of CHD–COPD co-morbidity compared with non-Hispanic White adults. The lower COPD prevalence among Black adults aligns with national data and may reflect differences in smoking patterns as well as underdiagnosis due to limited access to spirometry-based diagnostics (26). Accordingly, the observed lower co-morbidity in racial/ethnic minority groups should be interpreted with caution, as it may reflect underdiagnosis rather than true differences in disease burden (24, 34).

Insurance coverage was associated with higher odds of CHD–COPD co-morbidity (aOR = 8.31; 95% CI: 5.10–13.54), likely reflecting ascertainment bias. Insured individuals have greater access to clinical encounters where both conditions can be identified, a process requiring more sustained healthcare engagement than diagnosis of a single condition. This is consistent with the smaller associations observed for CHD (aOR = 2.18) and COPD (aOR = 1.82). In cross-sectional survey data, insurance status often serves as a proxy for healthcare access and diagnostic opportunity rather than underlying disease risk (24, 34); thus, this estimate should not be interpreted causally.

## Strengths and limitations

This study has several strengths. The use of nationally representative NHIS IPUMS data with pooled survey weights (2020-2024) enables robust population-level estimates while accounting for complex survey design. The analytic approach combining categorical and continuous year models, predictive margins, and sensitivity analyses strengthens inference by reducing reliance on a single modeling strategy. Also, focusing specifically on CHD, rather than broader CVD, improves outcome specificity. Lastly, the use of the large sample size (n = 150,852) provides adequate power despite the low prevalence of CHD–COPD co-morbidity.

This study also has limitations that must be acknowledged. First, both CHD and COPD were based on self-reported physician diagnosis, which is subject to recall bias and potential underdiagnosis, particularly among populations with limited healthcare access (24, 34). Second, NHIS excludes institutionalized populations and active-duty military personnel, which may limit generalizability. Third, the repeated cross-sectional design precludes causal inference, and observed associations should not be interpreted as causal. Fourth, the five-year study window may be insufficient to detect long-term trends in a relatively rare outcome, and 2020 data collection was disrupted by the COVID-19 pandemic, with the suspension of NHIS in-person interviews and a shift to telephone-based data collection. This transition led to reduced response rates, potentially affecting response patterns and healthcare utilization, and may have introduced nonresponse bias despite survey weighting adjustments. Finally, NHIS lacks clinical detail, including spirometry data, COPD severity, and CHD subtypes, which limits mechanistic and clinical subgroup analyses. Future studies that incorporate linkage to clinical records or use spirometry-confirmed COPD definitions would provide important refinements to the prevalence estimates reported here.

## Conclusion

This study provides the first nationally representative, five-year assessment of CHD-COPD co-morbidity among United States adults using pooled NHIS-IPUMS data. CHD-COPD co-morbidity was rare and remained stable from 2020 to 2024, with no significant temporal trend. COPD prevalence declined over time, whereas CHD prevalence remained unchanged. Smoking was the strongest modifiable risk factor, with current and former smokers exhibiting substantially higher odds of co-morbidity. Older age, male sex, lower income, and residence in the South were consistently associated with elevated co-morbidity risk, highlighting persistent sociodemographic disparities in cardiopulmonary health. These findings underscore the importance and need for disease-specific surveillance of cardiopulmonary multimorbidity and targeted prevention strategies, integrated smoking cessation, and focused interventions for high-risk populations, particularly older, lower-income, Southern-residing adults.

### Public health implications

Despite the low prevalence, CHD-COPD co-morbidity is a clinically important public health concern due to the high morbidity and mortality associated with both conditions. Strong associations with smoking, older age, lower income, lower educational attainment, and geographic region highlight key targets for prevention and intervention. Smoking cessation is the most critical strategy, as current and former smokers have five- to six-fold higher odds of co-morbidity. Integrating cessation support into routine COPD and CHD care, particularly for lower-income populations and residents of the Southern region, should be prioritized. Also, the observed socioeconomic and geographic disparities underscore the need for targeted prevention efforts and improved access to care, including expanded cessation services and community-based interventions to reduce inequities in exposure and treatment. Although prevalence was low and stable, affected individuals may experience substantial clinical and economic burden, supporting the need for integrated cardiopulmonary care and targeted screening in high-risk groups. Continued surveillance using longer-term and clinically validated data is needed to monitor trends and guide prevention strategies.

## Ethical considerations

This study utilized de-identified publicly available IPUMS dataset; therefore, it is exempt from UTHealth Houston institutional review board (IRB) approval.

## Data Availability

The original contributions presented in the study are included in the article/Supplementary Material, further inquiries can be directed to the corresponding author.
