# Supplementary material for: Coronary heart disease and chronic obstructive pulmonary disease prevalence and temporal trends among United States adults: a national population-based study
Source: Front Epidemiol. 2026 Jun 25;6:1840095. doi: 10.3389/fepid.2026.1840095 (PMC13346163; doi:10.3389/fepid.2026.1840095)
Supplement: Supplementary file 1 [file Table1.docx]

**Supplementary Table 1.** Survey weighted adjusted annual prevalence of CHD by year (predictive margins)

| **Year** | **Adjusted prevalence (%)** | **95% CI** |
| --- | --- | --- |
| 2020 | 1.68 | 1.31–2.15 |
| 2021 | 1.88 | 1.47–2.39 |
| 2022 | 1.69 | 1.31–2.17 |
| 2023 | 1.73 | 1.35–2.21 |
| 2024 | 1.65 | 1.28–2.12 |

**Supplementary Table 2**. Survey-weighted adjusted annual prevalence of COPD by year (predictive margins)

| **Year** | **Adjusted prevalence (%)** | **95% CI** |
| --- | --- | --- |
| 2020 | 3.53 | 2.92–4.26 |
| 2021 | 3.12 | 2.60–3.74 |
| 2022 | 3.28 | 2.71–3.96 |
| 2023 | 3.08 | 2.56–3.68 |
| 2024 | 2.81 | 2.32–3.40 |

**Supplementary Table 3:** Survey-weighted adjusted annual prevalence of CHD-COPD co-morbidity by year (predictive margins)

| **Year** | **Adjusted prevalence (%)** | **95% CI** |
| --- | --- | --- |
| 2020 | 0.35 | 0.25 – 0.49 |
| 2021 | 0.43 | 0.31 – 0.60 |
| 2022 | 0.40 | 0.28 – 0.56 |
| 2023 | 0.41 | 0.29 – 0.57 |
| 2024 | 0.43 | 0.31 – 0.60 |
